# Electrochemical Detection of Ultratrace Lead Ion through Attaching and Detaching DNA Aptamer from Electrochemically Reduced Graphene Oxide Electrode

**DOI:** 10.3390/nano9060817

**Published:** 2019-05-30

**Authors:** Su Hwan Yu, Chang-Seuk Lee, Tae Hyun Kim

**Affiliations:** Department of Chemistry, Soonchunhyang University, Asan 31538, Korea; shsh422@naver.com (S.H.Y.); eriklee0329@sch.ac.kr (C.-S.L.)

**Keywords:** reduced graphene oxide, G-quadruplex, lead ion, electrochemical aptasensor

## Abstract

This paper describes a simple strategy for the ultratrace level detection of Pb^2+^ ion based on G-quadruplex DNA and an electrochemically reduced graphene oxide (ERGO) electrode. First, ERGO was formed on a glassy carbon electrode (GCE) by the reduction of graphene oxide (GO) using cyclic voltammetry. Subsequently, a methylene blue (MB)-tagged, guanine-rich DNA aptamer (Apt) was attached to the surface of ERGO via π-π interaction, leading to the Apt-modified ERGO electrode. The presence of Pb^2+^ could generate the folding of Apt to a G-quadruplex structure. The formation of G-quadruplex resulted in detaching the Apt from the ERGO/GCE, leading to a change in redox current of the MB tag. Electrochemical measurements showed the proposed sensor had an exceptional sensitivity for Pb^2+^ with a linear range from 10^−15^ to 10^−9^ M and a detection limit of 0.51 fM. The sensor also exhibited high selectivity for Pb^2+^, as well as many other advantages, such as stability, reproducibility, regeneration, as well as simple fabrication and operation processes.

## 1. Introduction

The monitoring of lead ions (Pb^2+^) in the aquatic ecosystem is of great importance due to their harmful effect on the environment and human health [1,2,3]. Exposure to even a very low level of Pb^2+^ may cause serious human ailments such as reproductive, neurological, cardiac, and mental disorders [2,3]. Therefore, the rapid detection of Pb^2+^ at trace levels is of considerable significance to human health and environmental monitoring. Conventional methods have been used in the laboratory for detecting trace amounts of Pb^2+^ include atomic absorption/emission spectrometry, inductively coupled plasma mass spectrometry (ICP-MS) etc. [4,5,6,7]. However, these methods often suffer from the requirement of sophisticated equipment and bulky system size. Furthermore, the costly apparatus and the complex pretreatment steps limit their application for on-site and real-time monitoring. Anodic stripping voltammetry (ASV) [8] is also conventionally employed to detect Pb^2+^ which is relatively simple and cost effective compared with other conventional methods however still suffers from the low sensitivity for trace analysis. Thus, the challenge remains to develop new approaches for Pb^2+^ detection with high sensitivity, selectivity, cost-effectiveness, and simplicity in order to protect human health and the ecological environment. To meet this objective, many different electrochemical techniques have been proposed and proven to be promising tools for Pb^2+^ detection due to their high sensitivity, easy operation, rapid analysis, and ease of miniaturization, as well as the low cost of analysis [9,10,11]. Among them, the electrochemical aptasensors have attracted a great deal of attention owing to their exclusive advantages, such as high selectivity, simplicity and superior stability [11,12,13,14]. In recent years, for the aptasensing of Pb^2+^, functional oligonucleotides such as Pb^2+^-induced allosteric G-quadruplexes have become a powerful and convenient tool [12,15]. In order to develop a high-performance electrochemical aptasensor, one crucial factor is the applied materials to immobilize DNA aptamers. Graphene-related nanomaterials are predominantly attractive as materials for modifying electrodes in order to enable superior electrochemical sensing performance, owing to their inherent electrocatalytic nature, high adsorption performance and large surface area [16,17,18,19]. For the application of the sensor in the detection of real samples, the reusability of the electrode is a very critical factor. Although a few electrochemical aptasensors can be reused by regenerating the electrode, complex regeneration processes and the unsatisfactory reusability of the regenerated sensors make the use of the sensors in real samples difficult. Consequently, it is important to explore the regeneration of the electrochemical aptasensor. In this study, we developed a regenerative, sensitive and selective electrochemical aptasensor for Pb^2+^ detection based on electrochemically reduced graphene oxide (ERGO).

## 2. Materials and Methods

### 2.1. Materials

The DNA aptamer (Apt) was purchased from BIONEER Corporation (Daejeon, Korea) and its sequence is 5’-Methylene Blue-GGTGGTGGTGGTTGTGGTGGTGGTGG-3’. Graphite powder, phosphate buffer saline (PBS), Tris(hydroxymethyl)aminomethane hydrochloride (Tris-HCl), and potassium hexacyanoferrate (K_4_[Fe(CN)_6_]) were purchased from Sigma-Aldrich, Inc. (St. Louis, MO, USA); and 1,4,7,10-tetraazacyclododecane-1,4,7,10-tetraacetic acid (DOTA) was purchased from TCI Chemicals (Tokyo, Japan). All other chemicals were of analytical grade and used without further purification. Aqueous solutions were prepared with deionized water (>18 MΩ∙cm) obtained from a Millipore water purification system (MilliQ, specific resistivity > 18 MΩ∙cm, Millipore Korea, Co., Ltd. Seoul, Korea).

### 2.2. Instrumentation

All electrochemical measurements were performed with a Model 660D electrochemical analyzer/workstation (CH Instruments, Inc., Austin, TX, USA) using a conventional three-electrode cell. All measurements including cyclic voltammetry (CV), differential pulse voltammetry (DPV) and electrochemical impedance spectroscopy (EIS) were conducted at room temperature (25 °C) with a glassy carbon electrode (GCE), ERGO/GCE and Apt/ERGO/GCE as working electrodes, Ag/AgCl (3 M NaCl) reference and the Pt wire counter electrode in 10 mM PBS, or Tris buffer solution (pH 7.4). In CV experiments, the applied potential was scanned from −0.2 to 0.6 V (vs. Ag/AgCl) with scan rate of 10 mV/s and the sample interval of 1 mV. The parameters applied for DPV experiments were: scanning range between 0.1 to −0.5 V (vs. Ag/AgCl), amplitude of 0.05 V, pulse width of 0.2 s, and pulse time of 0.5 s. EIS measurements were carried out in 0.1 M Tris solutions containing 5 mM [Fe(CN)_6_]^4−^ at the formal potential of 0.222 V (vs. Ag/AgCl) with the AC voltage amplitude of 10 mV, and the voltage frequencies ranged (10^6^ to 10^−1^) Hz. The data obtained by EIS was fitted by using ZSimpWin 3.21 software (AMETEK. Inc., Oak Ridge, TN, USA). Raman spectroscopy was performed using an EnSpectr R532 Raman spectrometer (Enhanced Spectrometry, Inc., Torrance, CA, USA) with an excitation laser wavelength of 532 nm and laser power of 30 mW. The morphology of the fabricated electrode was examined using a scanning electron microscopy (SEM) system (JEOL, JSM 5600 LV, Tokyo, Japan).

### 2.3. Fabrication of Apt/ERGO/GCE Sensor

Graphene oxide (GO) was synthesized by a modified Hummers method [20]. The synthesized GO was then dispersed in 10 mM PBS buffer (pH 7.4) by ultrasonication for 1 h to form homogeneous yellow-brown GO solution (0.3 mg mL^−1^ in 10 mM PBS buffer, pH 7.4). Prior to the electrochemical fabrication of ERGO/GCE, GCE was carefully polished with 0.5 and 0.05 μm alumina powder to obtain a mirror-like surface, washed by sonication for 10 min, and rinsed with deionized water. ERGO/GCE was prepared using CV (0.8~−1.5 V vs. Ag/AgCl, 10 mV∙s^−1^, 3 cycles) in GO solution (0.3 mg∙mL^−1^ in 10 mM PBS buffer, pH 7.4) while using a GCE as a working electrode. Finally, Apt was immobilized onto the ERGO/GCE electrode. Briefly, the ERGO/GCE was incubated in 1 µM of the Apt solution containing 10 mM Tris buffer (10 mM Tris-HCl, 0.1 M NaCl, pH 7.4) for 5, 10, 20, 30, 40, 50, and 60 min, respectively. After the Apt-modified ERGO/GCE (Apt/ERGO/GCE) was washed with buffer and then dried at room temperature before it was ready to be used in the complete cell apparatus.

## 3. Results and Discussion

### 3.1. Charaterization of Apt/ERGO/GCE-Based Pb^2+^ Sensor

**Scheme 1:**
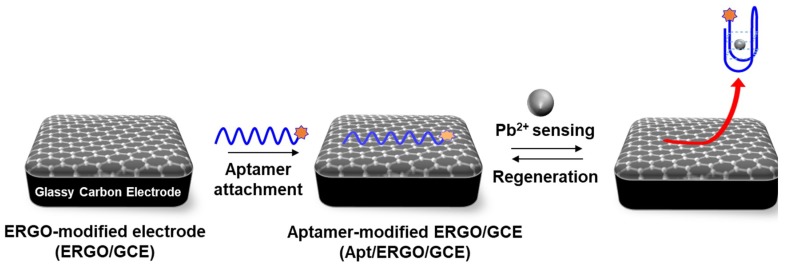
Schematic diagram depicting the fabrication of Ap/ERGO/GCE based electrochemical aptasensor for the detection of Pb^2+^.

In this work, we simply attached guanine-rich DNA aptamer onto the ERGO electrode via π-π stacking interaction to construct an electrochemical aptasensor which exhibited the ultrasensitive and highly selective detection of Pb^2+^ ions. Scheme 1 illustrates the overall procedure for the fabrication of the sensor and its detection for Pb^2+^; as can be seen, the method is facile, rapid, and efficient. First, ERGO was prepared and deposited on a GCE by the direct reduction of GO using CV, as reported previously [21,22,23]. Subsequently, methylene blue (MB)-labeled DNA aptamer with guanine-rich sequence (Apt) was immobilized onto the ERGO-modified GCE (EGRO/GCE) via π-π stacking interaction. Upon the addition of Pb^2+^, the Apt on the ERGO/GCE would undergo a conformational change to G-quadruplex due to the special induction capacity of Pb^2+^ to guanines [24]. This conformational change induced the Apt to be separated from the ERGO surface, which generates significant change in electrochemical signal of MB label. The regeneration of the sensor was possible by simply adding 1,4,7,10-tetraazacyclododecane-1,4,7,10-tetraacetic acid (DOTA) into the measurement solution after Pb^2+^ measurements. The proposed sensor features facile sensor fabrication and the easy implementation of analysis, along with ultrasensitivity, high selectivity, and easy regeneration.

**Figure 1:**
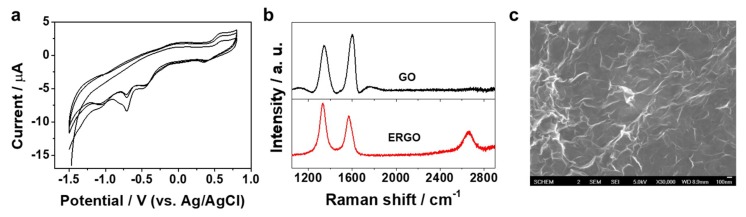
(**a**) CV curves recorded at GCE in 10 mM PBS buffer solution (pH 7.4) containing 0.3 mg mL^−1^ GO at a scan rate of 10 mV; (**b**) Raman spectra of GO and ERGO; (**c**) SEM image of ERGO formed on GCE.

Figure 1a shows typical CV curves recorded during the reduction of GO on GCE, revealing a characteristic behavior of GO electrolysis [23,25]. Irreversible cathodic waves around −0.46 and −0.7 V appeared and decreased in subsequent scans, which is due to the reduction of the oxygen functional groups of GO. This indicated GO was reduced and electrodeposited onto the GCE surface, leading to the formation of ERGO/GCE. Raman spectroscopic measurements confirm that significant structural changes occurred during the electrochemical reduction of GO to ERGO. As shown in Figure 1b, the Raman spectra obtained for GO exhibited the typical D band at 1348 cm^−1^ and G band at 1600 cm^−1^, corresponding to intervalley scattering of disordered structure and first-order scattering of the E_2g_ mode for sp^2^-hybridized carbon, respectively [26]. Compared with that of GO, the D band of ERGO shifted to a lower frequency at 1332 cm^-1^, indicating the reduction of GO to ERGO. Moreover, the intensity ratio of D and G bands (I_D_/I_G_) increased from 0.82 to 1.28 during the reduction of GO to ERGO, indicating a decrease in the average size of the sp^2^ carbon network by the removal of oxide functionalities [18]. Meanwhile, the Raman spectrum of GCE in Figure S1a (Supporting Information) also shows two pronounced peaks (D and G) which reveal the presence of small graphitic crystallites embedded in the amorphous matrix. Compared with the Raman spectrum of GCE, the adsorption of ERGO induced the appearance of a novel peak at 2663 cm^−1^, corresponding to 2D band which is related to the stacking nature of graphene layers. In addition, I_D_/I_G_ for GCE also is different from that of ERGO, indicating a complete coverage of the GCE surface by the ERGO film. The surface morphology of the ERGO/GCE was characterized by SEM. As shown in Figure 1c and Figure S1b (Supporting Information), the SEM images clearly confirm that a wrinkled ERGO film was formed and deposited on the GCE [20,21,22]. Taking advantage of a very stable coating and high surface area of the electrode, Apt molecules were immobilized onto the ERGO/GCE via π-π stacking to construct an Apt/ERGO/GCE-based electrochemical aptasensor for Pb^2+^.

**Figure 2:**
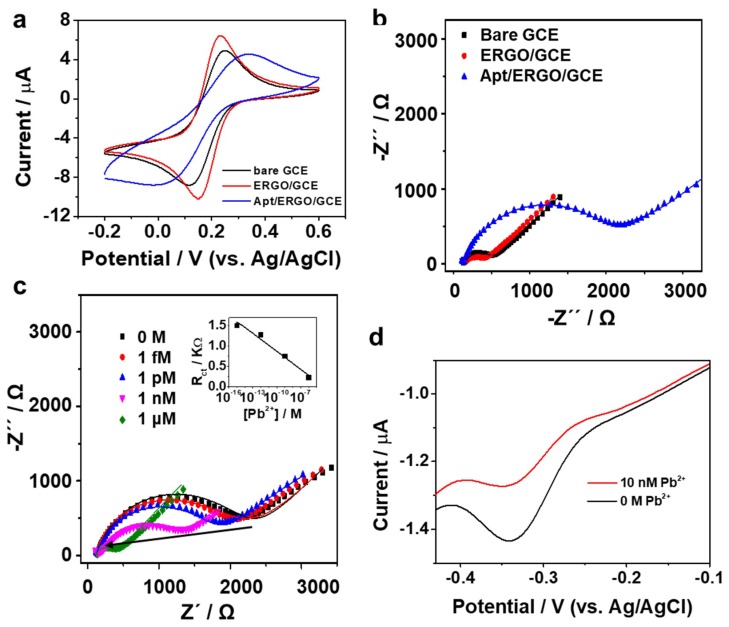
(**a**) CV curves recorded at a scan rate of 10 mV s^−1^ of GCE, ERGO/GCE, and Apt/ERGO/GCE in 10 mM Tris buffer containing 5 mM [Fe(CN)_6_]^4−^; (**b**) Impedance complex plane plots of GCE, ERGO/GC, and Apt/ERGO/GCE in 10 mM Tris buffer containing 5 mM [Fe(CN)_6_]^4−^; (**c**) Impedance complex plane plots for the Apt/ERGO/GCE immersed in different concentrations of Pb^2+^ (0 M, 1 fM, 1 pM, 1 nM, and 1 μM). Inset: the linear relationship between ΔR_ct_ and the concentrations of Pb^2+^ over the range of 1 fM–1 μM; (d) DPV curves of Apt/ERGO/GCE in the absence and presence of 10 nM Pb^2+^ in 10 mM Tris buffer at pH 7.4. The accumulation time for Apt (1 µM) coating was 20 min and the incubation time for Pb^2+^ detection was 20 min.

To explore the surface characteristics of the modified electrodes, the electron transfer kinetics of GCE, ERGO/GCE and Apt/ERGO/GCE were studied using voltammetric and impedance techniques utilizing Fe(CN)_6_^4−^ as a redox probe. Figure 2a shows the voltammetric responses of GCE, ERGO/GCE, and Apt/ERGO/GCE in 10 mM Tris buffer containing 1 mM Fe(CN)_6_^4−^., exhibiting a pair of well-defined redox peaks. Compared with the voltammetric behavior of GCE, the ERGO/GCE had a smaller peak-to-peak separation (ΔE_p_) with enhanced peak currents (I_p_), indicating a better electrochemical performance owing to the enhanced surface area and electrocatalytic property of ERGO. On the other hand, the Apt/ERGO/GCE showed a considerable decrease of I_p_ with an increase of ΔE_p_, suggesting sluggish electron transfer due to insulative Apt coating, which proved the successful formation of Apt/ERGO/GCE.

To better understand each construction step of the aptasensor, the electron transfer kinetics on GC, ERGO/GCE and Apt/ERGO/GCE was studied using EIS measurements utilizing Fe(CN)_6_^4−^ as a redox probe. Figure 2b shows the impedance complex plane plots presented as Nyquist plots in which a semicircle portion at higher frequencies corresponds to the charge transfer process. The charge transfer resistance (R_ct_) can be calculated from the diameter of the semicircle. With respect to the bare GCE, a decrease in the R_ct_ value was observed for the ERGO/GCE, suggesting the good conductivity owing to the high electroconductive nature of graphene materials. However, with the modification of Apt on ERGO/GCE, the resistance for Apt/ERGO/GCE increased significantly, which could be ascribed to the impeding effect of the DNA aptamer to the electron transfer. The fitted EIS data of Figure 2b are listed in Table S1. This implies that the Apt was successfully immobilized on the surface of the ERGO/GCE. These results confirm that all the fabrication steps were successfully performed. In order to test if the proposed sensing principle works well, a series of different concentrations of Pb^2+^ were measured by EIS. Figure 2c shows the impedance complex plane plots obtained using the Apt/ERGO/GCE after addition of different concentrations of Pb^2+^ in 10 mM Tris buffer containing 5 mM [Fe(CN)_6_]^4−^. Upon the addition of increasing concentrations of Pb^2+^ ion, the Apt/ERGO/GCE exhibited a gradual decrease in the R_ct_ value, and eventually, the addition of 1 μM Pb^2+^ made the resistance decrease to the value, comparable to that of the ERGO/GCE, which was due to the formation of Pb^2+^-induced allosteric G-quadruplex and thereby detachment of Apt from the electrode surface. The R_ct_ value from EIS exhibited linear relation toward logarithmic values of Pb^2+^ concentration in the range of 1 fM –1 μM as shown in Figure 2c inset. Parameter values obtained from fitting of the impedance plots are listed in Table S2. This verified the feasibility of the Apt/ERGO/GCE based aptasensor for the detection of Pb^2+^. Since Apt has an electroactive MB tag as electrochemical indicator, we expected the Apt/ERGO/GCE to exhibit the voltammetric sensing feature of Pb^2+^ when assuming that the addition of Pb^2+^ generates a conformational change of Apt to G-quadraplex, which can result in a voltammetric signal change. The voltammetric sensing behavior was examined by DPV. DPV is highly sensitive and fast method in very low concentrations of a redox probe and irreversible redox reaction, compared with other voltammetric techniques such as CV, linear sweep voltammetry (LSV), and square wave voltammetry (SWV). DPV parameters applied were according to those for our previous experiments with MB tagged DNA: amplitude of 0.05 V, pulse width of 0.2 s, and pulse time of 0.5 s [27]. Indeed, we confirmed the voltammetric sensing behavior of Pb^2+^ using the MB tag as an electrochemical indicator on the Apt/ERGO/GCE. Figure 2d shows the typical DPV curves of the MB tagged on the signal probe whose reduction peak is observed at −0.34 V. Upon the addition of 10 nM Pb^2+^, the Apt/ERGO/GCE exhibited a decrease in peak current (I_p_), due to an increase in the charge transfer barrier resulted from the detachment of the Apt from the ERGO/GCE, indicating the potential for voltammetric analysis using this sensor.

### 3.2. Optimization of Experimental Conditions

**Figure 3:**
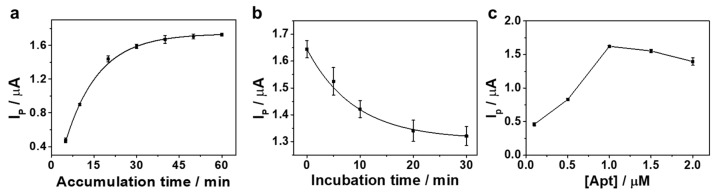
Effects of (**a**) the accumulation time for Apt immobilization ([Apt] = 1 µM), (**b**) the incubation time for Pb^2+^ reaction on the Apt/ERGO/GCE response signal ([Apt] = 1 µM, [Pb^2+^] = 10 nM, Apt accumulation time = 20 min), and (**c**) the concentration of Apt signal (Apt accumulation time = 20 min).

To obtain the best sensing performance, the influences of the accumulation time and Apt concentration for Apt immobilization, and the incubation time for Pb^2+^ sensing reaction on the voltammetric response of the resulting sensor in 10 mM Tris buffer were examined (Figure 3). The voltammetric response increased with the increasing time for the immobilization of the Apt up to 30 min; afterward the response was saturated (Figure 3a), implying the maximum immobilization of Apt on ERGO/GCE surface. Thus, 30 min was selected for accumulation time for sensor preparation. In addition, with the increasing incubation time for Pb^2+^ detection, the voltammetric response increased and tended to a steady value at 20 min (Figure 3b). Thus, 20 min was enough for the reaction. As shown in Figure 3c, for an Apt concentration, we found the best analytical performance in 1 µM. Therefore, we optimized the working conditions of electrochemical sensing for the next application.

### 3.3. Electroanalytical Performance of Apt/ERGO/GCE-Based Pb^2+^ Sensor

**Figure 4:**
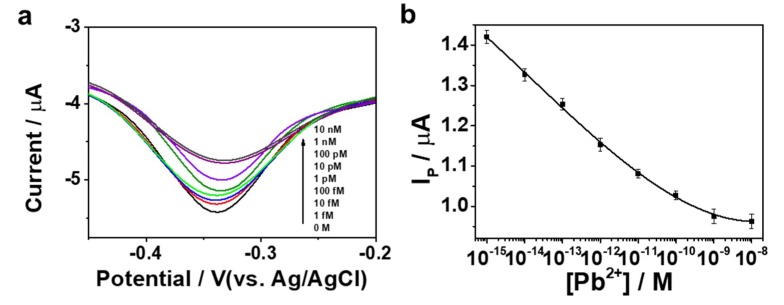
(**a**) DPV curves of Apt/ERGO/GCE sensor upon the addition of different concentrations of Pb^2+^ in 10 mM Tris buffer; (**b**) The corresponding calibration curve for Apt/ERGO/GCE sensor with peak current response versus Pb^2+^ concentration.

Under the optimized experimental conditions, we investigated the sensitivity of the proposed aptasensor toward Pb^2+^. Figure 4a displays the DPV curves of the Apt/ERGO/GCE after the addition of different concentrations of Pb^2+^. A dramatic decrease and then saturation in the peak current (I_p_) was observed with the increasing concentrations of Pb^2+^. By measuring I_p_ of Apt/ERGO/GCE upon adding different concentrations of Pb^2+^, we obtained the calibration curve for Pb^2+^ concentrations ranging from 10^−15^ to 10^−8^ M (Figure 4b). Figure 4b reveals the linear responses to the concentration of Pb^2+^ over the range of 10^−15^–10^−9^ M. A detection limit of 0.51 fM for Pb^2+^ ions was estimated based on S/N = 3. As compared with common methods and other DNA-based electrochemical sensors for Pb^2+^ (Table 1 and Table 2), our sensor shows a lower detection limit with a wider dynamic range than most of the previously reported methods for Pb^2+^. In addition, the cost-effectiveness of our sensor is superior to conventional methods. The initial acquisition cost of our sensor could be much lower between $150 and $500 than those of other optical conventional methods between $500 and $2000.

**Figure 5:**
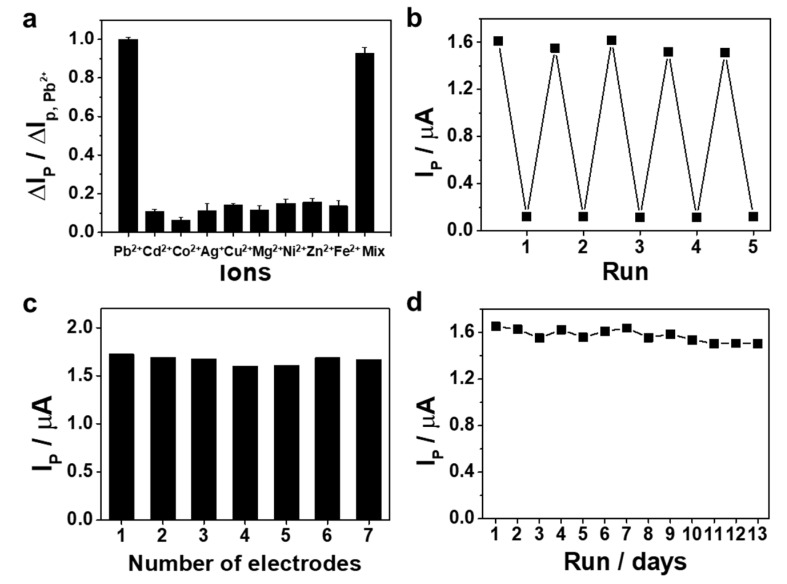
(**a**) Selectivity of the proposed sensor over other competing metal ions. The concentrations of Pb^2+^ and other metal ions are 1 pM and 10 nM, respectively. Mixed solution (Mix) contained 1 pM Pb^2+^ and various metal ions (Cd^2+^, Co^2+^, Ag^+^, Cu^2+^, Mg^2+^, Ni^2+^, Zn^2+^, Fe^2+^) of 10 nM.; (**b**) DPV peak currents of the sensor in 5 response and regeneration runs; (**c**) Reproducibility of the sensor for seven different modified electrodes; (**d**) Stability of the sensor in thirteen days.

To test the selectivity of the sensor for Pb^2+^ detection, we performed the control experiments in the presence of various metal ions (Cd^2+^, Co^2+^, Ag^+^, Cu^2+^, Mg^2+^, Ni^2+^, Zn^2+^, and Fe^2+^). As shown in Figure 5a and Figure S2 (Supporting Information), among these metal ions only Pb^2+^ results in an obvious change in the voltammetric response, while other metal ions do not result in any discernible voltammetric change, indicating that our sensor exhibits a good selectivity to Pb^2+^ over other competing metal ions. We further tested the sensor response to Pb^2+^ in the mixed solution of various metal ions. The results indicated that the response of the sensor to Pb^2+^ was almost unaffected by the coexistence of other metal ions, even the concentration of interference ions is 10^4^ times higher than that of Pb^2+^. These results indicated that the sensor possesses an excellent selectivity to Pb^2+^. For the practical application of the sensor, additional analytical features, such as the regeneration, reproducibility, and stability of the Apt/ERGO/GCE were further evaluated. The electrochemical aptasensor was ready to be regenerated by adding DOTA solution. As shown in Figure 5b, after being challenged with 1 nM of Pb^2+^ and then reacted with 1 mM DOTA for 10 min, the aptasensor was regenerated for more than five cycles. The regeneration of the aptasensor was regulated by strong binding of Pb^2+^ with DOTA [38] and subsequent conversion of Apt from its folded quadruplex to unfolded conformation, leading to its attachment onto the ERGO surface. The reproducibility and stability of the sensor were tested in 10 mM Tris buffer, as shown in Figure 5c,d. The reproducibility was evaluated by comparing the voltammetric responses produced by different electrodes under the same conditions of preparation. Figure 5c shows the peak current values of seven different modified electrodes, indicating the response of all fabricated sensors is highly reproducible. Moreover, the sensor was stored under atmosphere condition for 13 days to investigate its stability. As shown in Figure 5d, no noticeable change in I_p_ was observed, compared with the initial current response after 13 days. The above sensing performances suggest the suitability of the Apt/ERGO/GCE sensor for their practical use to real samples. The application of the sensor in real samples was indeed demonstrated with promising reproducibility and reproducibility. As shown in Table 3, the results obtained for tap water, valley water, and secondary treated wastewater samples showed good recovery values, suggesting the acceptability of Apt/ERGO/GCE electrodes for the practical analysis of Pb^2+^ in environmental samples. Therefore, these results indicate that our sensor completely met the selective requirements for environmental applications.

## 4. Conclusions

We report a facile strategy for the fabrication of an ultrasensitive electrochemical Pb^2+^ aptasensor using T-rich, MB-tagged DNA (Apt) and ERGO-modified GCE. This approach utilizes π-π binding interaction as an electrode modification between Apt and ERGO surface, leading to Apt-modified ERGO/GCE. In our sensing strategy, the presence of Pb^2+^ causes a conformation change of the Apt to a folded G-quadruplex structure, leading to the separation of Apt from the ERGO surface. This attaching and detaching phenomena of Apt on ERGO/GCE enabled the ultratrace level detection of Pb^2+^ with high selectivity. This sensor possesses some attractive features, such as simple electrode fabrication, facile operational convenience, stability, and reproducibility. In addition, the sensor can be easily reused by simply adding DOTA to the measurement solution. These remarkable features would endow the proposed sensor with great promise in a wide range of applications to the environmental and biomedical fields.

## Figures and Tables

**Table 1:** Comparison of the present work with different methods for determination of Pb^2+^.

Methods	Detection Limit	Linear Range (M)	Ref.
ICP-MS	0.38 nM	-	[28]
ICP-AES	40 nM	-	[29]
Fluorescence	0.3 pM	1.0 × 10^−12^–3.0 × 10^−6^	[30]
Colorimetry	20 pM	5 ×10^−11^–5 × 10^−9^	[31]
SERS	8.9 pM	10^−11^–10^−6^	[32]
ASV	3.9 nM	2.4 × 10^−10^–2.9 × 10^−7^	[8]
DPV	0.51 fM	10^−15^–10^−9^	This work

**Table 2:** Comparison of the present work with previously reported electrochemical aptasensors for Pb^2+^.

**Electrode Materials**	**Detection Limit**	**Linear Range (M)**	**Ref.**
DNAzyme/Au	300 nM	5 × 10^−7^–10^−5^	[14]
HP/AuNPs/SPCE	34 pM	5 × 10^−11^–2 × 10^−7^	[33]
DNAzyme/AuNP/Au	100 pM	10^−10^–10^−7^	[34]
OMC–GNPs/L-Lys/GCE	200 pM	5 × 10^−10^–5 × 10^−5^	[35]
DNAzyme/depAu/GCE	33 fM	10^-13^–2 × 10^−7^	[36]
TSP/Au	80 pM	10^−11^–10^−6^	[37]
primer-AuNPs-S3/Pb^2+^ /MCH/S2/S1/depAu/GCE	3.3 fM	10^−15^–10^−11^	[13]
Apt/ERGO/GCE	0.51 fM	10^−15^–10^−9^	This work

**Table 3:** Determination of Pb^2+^ at various concentrations in tap water.

Sample	Added (M)	Found (M)	RSD (%)	Apparent Recovery Factors (%)
Tap water	-	Not detected	-	-
	1.00 × 10^−15^	0.98 × 10^−15^	2.04	98
	1.00 × 10^−12^	1.10 × 10^−12^	2.57	110
	1.00 × 10^−9^	1.03 × 10^−9^	2.41	103
Valley water	-	Not detected	-	-
	1.00 × 10^−15^	0.97 × 10^−15^	1.82	97
	1.00 × 10^−12^	0.95 × 10^−12^	2.55	95
	1.00 × 10^−9^	1.03 × 10^−9^	1.85	103
Secondary treated wastewater ^a^	-	Not detected	-	-
	1.00 × 10^−15^	1.05 × 10^−15^	1.99	105
	1.00 × 10^−12^	1.03 × 10^−12^	1.27	103
	1.00 × 10^−9^	1.05 × 10^−9^	1.85	105

^a^ Secondary treated wastewater was obtained from the Environment Management Corporation (Asan, Korea).

## Supplementary Materials

The following are available online at https://www.mdpi.com/2079-4991/9/6/817/s1.
