# Supplementary material for: Electrochemical Detection of Ultratrace Lead Ion through Attaching and Detaching DNA Aptamer from Electrochemically Reduced Graphene Oxide Electrode
Source: Nanomaterials (Basel). 2019 May 30;9(6):817. doi: 10.3390/nano9060817 (PMC6630585; doi:10.3390/nano9060817)
Supplement: Supplementary file 1 [file nanomaterials-09-00817-s001.pdf]

Supporting Information

Electrochemical Detection of Ultratrace Lead Ion through Attaching and Detaching DNA Aptamer from Electrochemically Reduced Graphene Oxide Electrode

Su Hwan Yu†, Chang-Seuk Lee†, and Tae Hyun Kim*

Department of Chemistry, Soonchunhyang University, Asan 31538, Korea; shsh422@naver.com (S.H.Y.) ; eriklee0329@sch.ac.kr (C.-S.L.)

* Correspondence: thkim@sch.ac.kr; Tel.: +82-41-530-4722

† These authors contributed equally to this work.

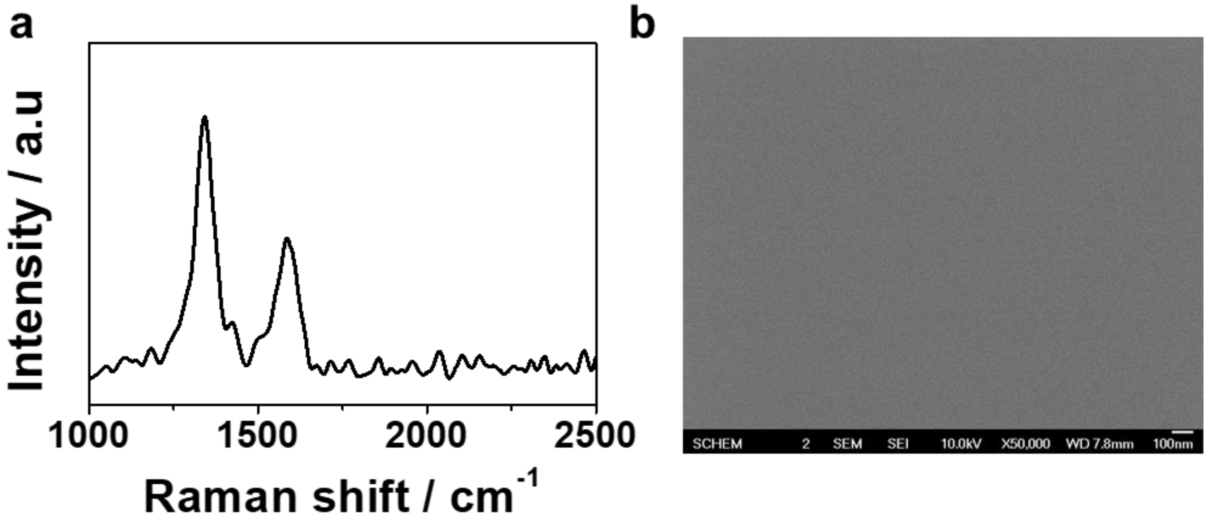

Figure S1. (**a**) Raman spectra of GCE; (**c**) SEM image of GCE.

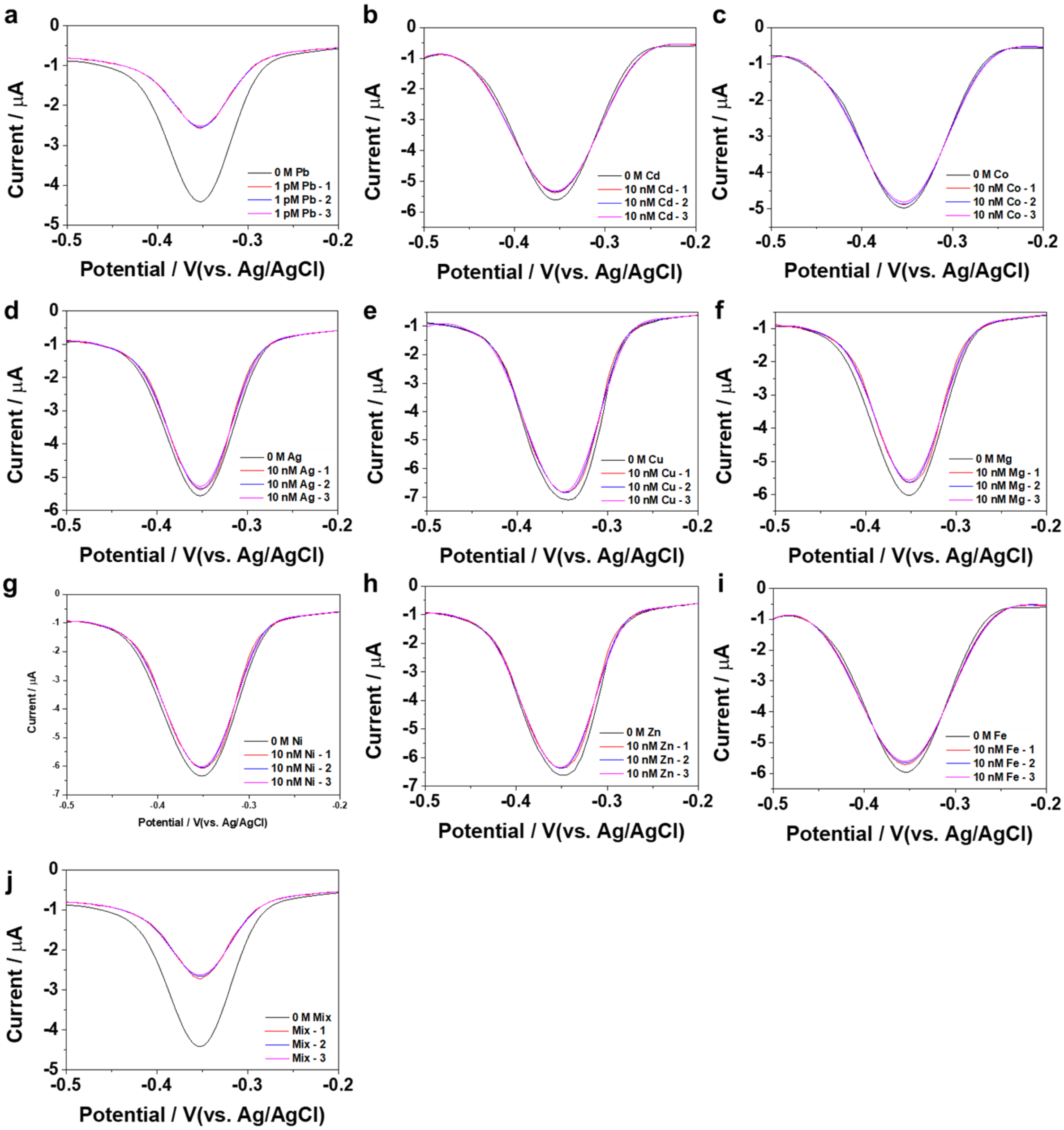

Figure S2. DPV curves of Apt/ERGO/GCE in the absence and presence of (a) 1 pM Pb^{2+}, (b) 10 nM Cd^{2+}, (c) 10 nM Co^{2+}, (d) 10 nM Ag^{+}, (e) 10 nM Cu^{2+}, (f) 10 nM Mg^{2+}, (g) 10 nM Ni^{2+}, (h) 10 nM Zn^{2+}, (i) 10 nM Fe^{2+}, and (j) mixed solution in 10 mM Tris buffer at pH 7.4. Mixed solution (Mix) contained 1 pM Pb^{2+} and various metal ions (Cd^{2+}, Co^{2+}, Ag^{+}, Cu^{2+}, Mg^{2+}, Ni^{2+},

Zn^{2+}, Fe^{2+}) of 10 nM. Each measurement was performed three times. The accumulation time for Apt (1 μM) coating was 20 min and the incubation time for Pb^{2+} detection was 20 min.

Table S1. Parameters values obtained from fittings of the impedance plots represented in Figure 2b.

**EIS plot**	**R_{ct}(Ω)**	**C(μF)**
Bare GCE	467.28	0.35
ERGO/GCE	336.68	0.24
Apt/ERGO/GCE	2077.49	1.74

Table S2. Parameters values obtained from fittings of the impedance plots represented in Figure 2c.

**Concentration**	**R_{ct}(Ω)**	**C(μF)**
0 M	2077.49	1.74
1 fM	1737.12	1.63
1 pM	1530.82	1.19
1 nM	1392.62	0.77
